# CARD-Associated Risk Score Features the Immune Landscape and Predicts the Responsiveness to Anti-PD-1 Therapy in IDH Wild-Type Gliomas

**DOI:** 10.3389/fcell.2021.653240

**Published:** 2021-03-19

**Authors:** Depei Li, Wanming Hu, Xiaoping Lin, Ji Zhang, Zhenqiang He, Sheng Zhong, Xia Wen, Peiyu Zhang, Xiaobing Jiang, Hao Duan, Chengcheng Guo, Jian Wang, Jing Zeng, Zhongping Chen, Yonggao Mou, Ke Sai

**Affiliations:** ^1^Department of Neurosurgery/Neuro-oncology, Sun Yat-sen University Cancer Center, Guangzhou, China; ^2^State Key Laboratory of Oncology in South China, Collaborative Innovation Center for Cancer Medicine, Sun Yat-sen University Cancer Center, Guangzhou, China; ^3^Department of Pathology, Sun Yat-sen University Cancer Center, Guangzhou, China; ^4^Department of Nuclear Medicine, Sun Yat-sen University Cancer Center, Guangzhou, China

**Keywords:** IDH, gliomas, CARD, tumor immune microenvironment, checkpoint immunotherapy

## Abstract

**Background:**

Proteins containing the caspase recruitment domain (CARD) play critical roles in cell apoptosis and immunity. However, the impact of CARD genes in tumor immune cell infiltration, responsiveness to checkpoint immunotherapy, and clinical outcomes of gliomas remains unclear. Here, we explore using CARD genes to depict the immune microenvironment and predict the responsiveness of gliomas to anti-PD-1 therapy.

**Methods:**

The genome and transcriptome data of 231 patients with isocitrate dehydrogenase wild-type (IDH-wt) gliomas were retrieved from The Cancer Genome Atlas (TCGA) database to screen CARD genes associated with T lymphocyte infiltration in gliomas. Weighted co-expression network and LASSO penalized regression were employed to generate a CARD-associated risk score (CARS). Two independent and publicly available datasets were used to validate the effectiveness of CARS.

**Results:**

The CARS divided the 231 glioma patients into high- and low-risk subgroups with distinct immune microenvironment and molecular features. The high-risk group had high CARS and was characterized by enrichment of dysfunctional T lymphocytes in a profound immunosuppressive microenvironment, whereas the low-risk group had low CARS and exhibited an immune exclusion genotype. Moreover, signaling aberrations including upregulation of PI3K/Akt/mTOR, NF-κB, and TGF-β were found in the high-risk group. In contrast, the activated WNT pathway was more evident in the low-risk group. Furthermore, we found that an elevated CARS indicated a decreased overall survival for IDH-wt gliomas under standard care but a clinical benefit from checkpoint immunotherapy.

**Conclusion:**

This study developed an immune- and prognosis-relevant risk score, which could be used to enhance our understanding of the heterogeneity of immune microenvironment of gliomas and facilitate to identify patients who will benefit from checkpoint immunotherapy.

## Background

Glioma represents one of the most prevalent primary brain tumors in adults (Alexander and Cloughesy, 2017). Mutation in isocitrate dehydrogenase (IDH) is a critical genetic alteration and a key biomarker for pathological classification and prognosis in gliomas (Chen et al., 2012). IDH mutation is identified in the majority of low-grade gliomas (LGG) and generally predicts an indolent clinical course. In contrast, primary glioblastoma multiforme (GBM) as well as a subset of LGGs are IDH-wt manifest with an aggressive behavior and therapeutic resistance. The prognosis of IDH-wt gliomas remains disappointing. The median overall survival (OS) of GBMs is only 20.5 months regardless of intensive treatment (Stupp et al., 2015). Meanwhile, IDH-wt LGGs exhibit molecular and clinical traits similar to GBMs and have much worse prognosis compared to the IDH-mutated counterparts (Reuss et al., 2015). The management of IDH-wt gliomas is challenging and novel therapies are needed.

Checkpoint blockade immunotherapy revolutionized the treatment landscape across various types of solid cancers, and therefore generated interests in glioma (Waldman et al., 2020). Unfortunately, the success of immune checkpoint inhibition (ICI) in other malignancies has not been reproduced in gliomas. Despite the promising preclinical results, some phase III clinical trials of ICI with Nivolumab failed to yield significant survival benefit in unselected recurrent or newly diagnosed GBM (Dunn et al., 2020; Reardon et al., 2020). The heterogeneity of tumor immune microenvironment in gliomas may account for these clinical outcomes. Dissecting the intrinsic properties that confer the immunosuppression and identifying predictive biomarkers for the responsiveness to anti-PD-1 therapy is of paramount necessity.

The caspase recruitment domain (CARD), a member of the death domain family, is a critical protein interaction module (Park, 2019). More than 30 CARD-containing proteins have been identified in human. Functioning as a homotypic protein interaction motif, CARD orchestrates the networks of proteins through CARD–CARD interaction (Damiano and Reed, 2004). CARD was originally found in a subset of apoptotic proteins such as caspase-2, -9, Apaf-1, and cIAP-1. The CARD-containing caspases interact with their adaptor molecules to trigger the apoptotic cascade through facilitating the assembly of the apoptosome (Bouchier-Hayes and Martin, 2002). In addition to the activation of apoptosis, accumulating evidence suggests that CARD-containing proteins also function as scaffold components in a variety of signaling pathways crucial for immune response (Matusiak et al., 2015). Caspase-1, a CARD-containing protease, is essential for the maturation of secreted IL-1β and plays a crucial role in inflammatory and immunogenic cell death (Miao et al., 2011). PYCARD, also known as apoptosis-associated speck-like protein containing a CARD (ASC), functions as an adaptor to bridge sensor proteins and effector molecules such as procaspase-1 within the inflammasome complex. The activation of inflammasome may be crucial for vaccine-induced humoral and cell-mediated immune responses. It has been reported that CD4+ T cells deficient for PYCARD exhibit impaired proliferative responses and a suppressive cytokine profile (Narayan et al., 2011). To date, the immune and clinical relevance of CARD genes and associated signaling in gliomas remains unclear.

Here, we leveraged the transcriptome expression data from the TCGA database to identify CARD genes that are associated with T lymphocyte infiltration in glioma. A CARD-associated risk score (CARS) was developed by using co-expression network and penalized regression. CARS stratified IDH-wt gliomas into two groups with distinct distribution of immune cell infiltration and molecular features. The effectiveness of CARS was validated by two other independent cohorts. Importantly, we found that an elevated CARS was associated with an unfavorable outcome of glioma patients under standard care but a survival benefit from anti-PD-1 immunotherapy. Our findings may facilitate to identify glioma patients who are potentially benefited from checkpoint immunotherapy.

## Materials and Methods

### Data Extraction and Grouping

Gene expression data [read counts and transcripts per million (TPM) values] and corresponding clinical information of glioma patients were downloaded from UCSC Xena browser: Toil RNA-seq recomputed project and TCGA Pan-Cancer cohort^1^ (Vivian et al., 2017). Among these, 231 IDH-wt glioma samples with survival data were retrieved for further analyses. Two independent datasets (CGGA and GSE16011) were used for validation (Gravendeel et al., 2009; Bao et al., 2014). RNA sequencing data (FPKM value) of gene expression was downloaded from the Chinese Glioma Genome Atlas (CGGA) database^2^. Then FPKM value was transformed into TPM values. Affymetrix microarray-based gene expression matrix with RMA normalization was downloaded from the Gene Expression Omnibus (GEO) database (accession: GSE16011^3^) (Gravendeel et al., 2009). The clinical information of these three cohorts is summarized in Supplementary Table 1.

### Transcriptomic and Genomic Data Processing

Genes were first filtered to include only protein-coding genes, and gene expressions were omitted when TPM values were less than 0.01. Genes with null expression in more than 75% of samples were removed, followed by removal of genes with median TPM expression <0.1. The filtered TPM values were then log_2_ transformed and normalized to the mean and standard deviation among the samples (*z* score transformation). *z* score transformation was also performed in microarray-based expression data across samples. Subsequent gene-expression analyses were implemented with the normalized TPM values. Somatic mutation and copy-number variation were estimated and visualized by using the TCGA OncoGrid algorithm. Tumor mutation burden (TMB) was represented by the sum of the number of non-synonymous mutations. Mutation rate, aneuploidy score, and neoantigen count of glioma samples were obtained from Thorsson’s study (Thorsson et al., 2019).

### Protein–Protein Interaction (PPI), Unsupervised Clustering, and Principal Component Analyses (PCAs)

Principal component analyses was implemented by using software R package *ade4* with the normalized TPM values. Unsupervised clustering analysis was performed by using *ConsensusClusterPlus* package and k-means method with 80% item resampling and 1,000 times repetition (Wilkerson and Hayes, 2010). The cumulative distribution function and consensus heatmap were used to determine the optimal *K*-value. PPI network was constructed by using the STRING online tool^4^.

### Differential Gene-Expression and Gene Set Enrichment Analyses

RNA-seq read counts were employed for DGE analysis by using *edgeR* package (Robinson et al., 2010). Immport database^5^ was used for immune function annotation of differentially expressed genes. A ranked list of genes created using −log10(P) × sign(log2(fold change)) (Reimand et al., 2019) from *edgeR* DGE output was conducted to generate enrichment scores by using the Preranked module of GSEA software (vision 4.1) (Subramanian et al., 2005) and C2: Canonical Pathway gene sets from the MSigDB database (vision 7.1) (Liberzon et al., 2011) with 1,000 permutations. The resulting enriched gene sets, with normalized enrichment score (NES) >2 and FDR < 0.01, were visualized in an enrichment map using Cytoscape (vision 3.7.2) software as described previously (Reimand et al., 2019). Fold change to median TPM value of each gene was calculated, then log2 transformed, and used for single-sample GSEA (ssGSEA), implemented in the *GSVA* package (Hänzelmann et al., 2013). ssGSEA enrichment score was calculated using gene sets composed of transcripts characteristic of an established T cell signature (Spranger et al., 2015), overall lymphocyte infiltration (Calabrò et al., 2009), immune cell populations (Charoentong et al., 2017), and signaling pathways (Liberzon et al., 2011). The enrichment scores generated by ssGSEA analysis were used to represent the relative abundance of immune cell or activity of signaling in each sample. CIBERSORT algorithm and signature matrix “LM22” were also used to deconvolve immune cell fractions (Newman et al., 2019). Cytotoxic activity (Davoli et al., 2017), MHC class-I (Lauss et al., 2017), and Batf3-dendritic cell (Batf3-DC) (Spranger et al., 2017) were calculated by taking mean expressions of genes in the gene sets. All gene sets we utilized are presented in Supplementary Table 2.

### Development of a CARS

Weighted gene co-expression network analysis (WGCNA) was done by using *WGCNA* package to find modules of highly correlated genes (Langfelder and Horvath, 2008). Gene modules associated with both the CARD cluster and lymphocyte infiltrations were selected. Hub genes in the selected modules were employed to conducting LASSO penalized Cox proportional hazards regression by using *glmnet* package for identifing genes with best prognostic significance. The linear combination of gene expressions weighted by the estimated regression coefficients in the multivariate model was used to calculate the risk scores of each patient.

### Tissue Microarray and Immunohistochemistry

Tissue microarrays (TMAs) were constructed by using paraffin-embedded specimens from 103 IDH-wt glioma patients who were treated at the Sun Yat-sen University Cancer Center (SYSUCC) from 2010 to 2016. Patient informed consent was obtained, and the study was approved by the Ethics Committee of the Sun Yat-sen University Cancer Center. TMAs were stained with PTX3 (Abcam, Cambridge, United Kingdom) by immunohistochemistry (IHC) method as previously described (Hu et al., 2017). IHC scoring criteria were established by combining the positive proportion (1 for 0–25%, 2 for 26–50%, 3 for 51–75%, 4 for >75%) and staining intensity (0 for no staining,1 for light yellow, 2 for yellowish brown, 3 for brown) of the stained tumor cells. The final PTX3 expression score (0–12) was achieved by positive ratio × staining intensity. The best cutoff value of PTX3 staining was determined by using ROC curves with respect to OS, and applied for developing a two-level grade system of PTX3 expression. All the samples were scored separately by two independent neuropathologists, who were blinded to the patient data. The discrepancies were resolved by consensus under a microscope for multi-viewing.

### Prediction of Survival Benefit From ICI Therapy

Genomic, transcriptomic, and clinical data of GBM patients underwent ICI treatment was obtained from a recently published study by Zhao et al. (2019) IDH-wt gliomas with resected tissues obtained after recurrence were included for analysis. Median risk score, PD-L1 expression, and TMB were investigated for prognostic prediction.

### Statistical Analysis

Normalized gene expressions and ssGSEA scores between two groups were compared by Wilcoxon tests. Categorical variables were compared by chi-squared or Fisher Exact test. Kaplan–Meier analyses with log-rank tests were performed to assess survival differences. Univariate and multivariate Cox regression were conducted to calculate hazard ratio (HR). Time-dependent ROC curve was depicted for OS prediction with *survivalROC* package. *R* (vision 3.6.2) and GraphPad Prism (version 8.0.1) software were applied for the statistical analyses. *P* < 0.05 was considered statistically significant.

## Results

### The CARD Genes Are Associated With T Lymphocyte Infiltration in IDH-wt Gliomas

**FIGURE 1:**
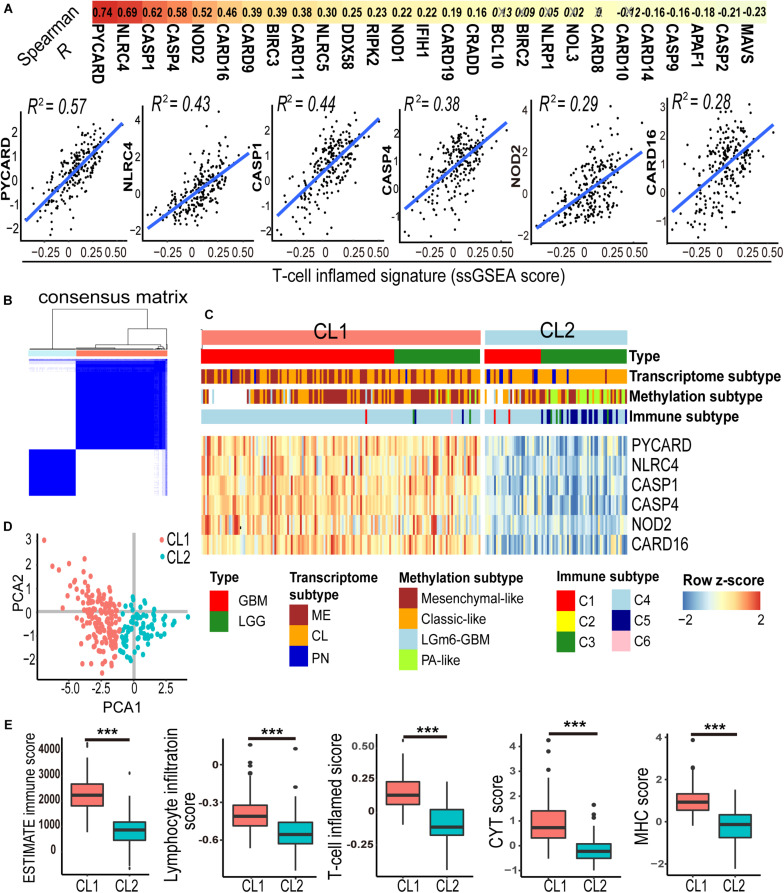
A panel of six-CARD genes associates with T lymphocyte infiltration in TCGA IDH wild-type gliomas. **(A)** Correlation between CARD gene expression and T-cell inflamed ssGSEA score. Top depicts Spearman *R* values. Non-significant correlations are crossed out. Scatter plots show fitted coefficient *R*^2^ values. **(B,C)** Consensus clustering matrix and heatmap with the six-CARD genes identified two clusters (CL1 and CL2). Histological and molecular subtypes were annotated for each patient. **(D)** PCA of two clusters. **(E)** Comparisons of immune score (from ESTIMATE), lymphocyte infiltration and T cell inflamed scores (from ssGSEA), and CYT and MHC scores (from mean gene expressions) between two clusters. *P* values: ***, <0.001.

Previous studies from other cancer types suggested that the tumor-infiltrating lymphocytes, particularly activated T lymphocytes (T cell inflamed genotype), are linked to prognosis and response to checkpoint immunotherapy (Olson and Luke, 2019). To determine the association between CARD-containing genes and T lymphocyte infiltration in glioma, we analyzed the expression profiles of 31 known CARD genes and correlated them with an established T cell-inflamed score (Spranger et al., 2015) using the ssGSEA method in these 231 IDH-wt glioma patients from the TCGA dataset. Six CARD genes, namely, *PYCARD*, *NLRC4*, *CASP1*, *CASP4*, *NOD2*, and *CARD16*, were found to be associated with the T cell inflamed metrics (Figure 1A, Spearman *R* > 0.4, *P* < 0.001). K-means clustering based on the expressions of the six CARD genes identified two main clusters (CL1 and CL2) in the IDH-wt gliomas (Figures 1B,C). PCA revealed pronounced difference in the expression portraits between the two clusters (Figure 1D). ESTIMATE algorithm (Yoshihara et al., 2013) showed elevated immune cell infiltration in CL1 (Figure 1E). We then evaluated the infiltration of lymphocytes and T cells using five scoring systems, including ESTIMATE immune score, Lymphocyte infiltration score, T cell inflamed score, CYT score, and MHC score. We demonstrated that the CL1 cluster had an enrichment of lymphocytes and T cells with increased immunological activities including cytolysis and antigen presentation (Figure 1E).

### The CARD Genes Are Associated With the Activation of Immune-Modulating Pathways

**FIGURE 2:**
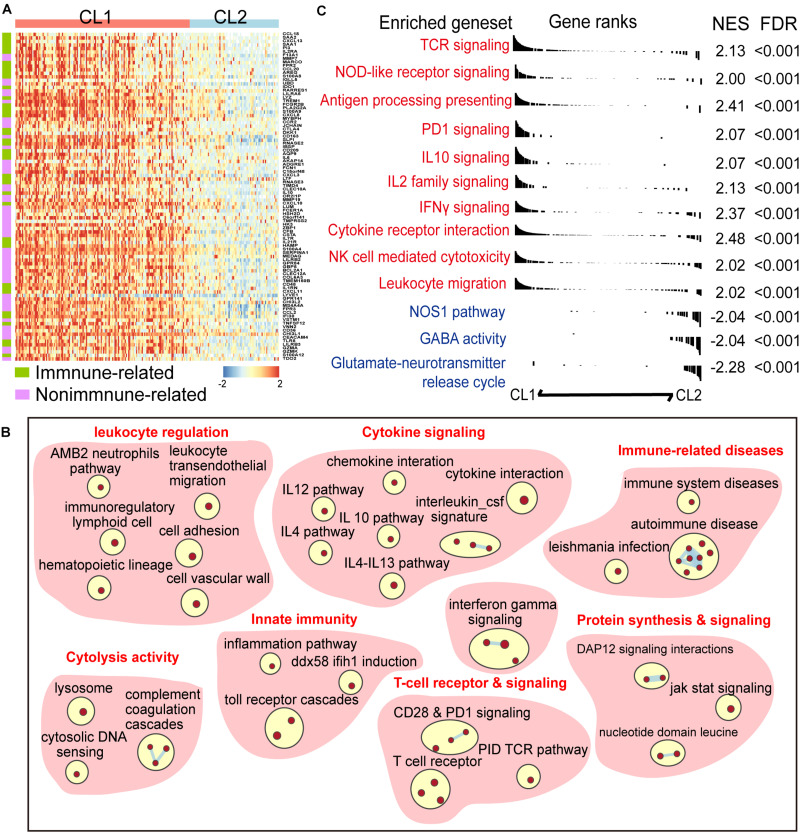
A panel of six-CARD genes associates with activation of immune pathway. **(A)** Top upregulated genes in CL1 versus CL2 (log_2_ fold change >2 and FDR < 0.01). Genes were sorted by the fold change and annotated using Immport database. **(B)** Overview of gene sets enriched in CL1 versus CL2 [normalized enrichment score (NES) >2 and FDR < 0.01]. **(C)** Differences in canonical gene sets between CL1 and CL2.

To explore the difference of gene expression pattern and activation of signaling pathways between the two clusters, we compared the transcriptional profiles by using differential gene-expression (DGE) analysis. A total of 814 and 987 transcripts were identified to be up- and downregulated in CL1, respectively (| log_2_ Fold change| > 1 and FDR < 0.01, Supplementary Table 3). We found that many of the top upregulated genes in CL1 had well-known immune-related functions in the ImmPort database annotation (Figure 2A and Supplementary Table 3), indicating differences in immune modulation between the two clusters. Meanwhile, Gene set enrichment analysis (GSEA) was performed, and it revealed a prominent enrichment of genes in key immune-related processes such as T cell receptor and signaling, innate immunity, and cytolysis activity in CL1. Activation of immune-stimulating pathways (e.g., IFN-γ and NOD-like receptor signalings) and those related with immune escape (e.g., PD-1 and IL-10 signalings) were observed in CL1 (Figures 2B,C and Supplementary Table 4).

### A CARS Predicts the Prognosis of IDH-wt Gliomas

**FIGURE 3:**
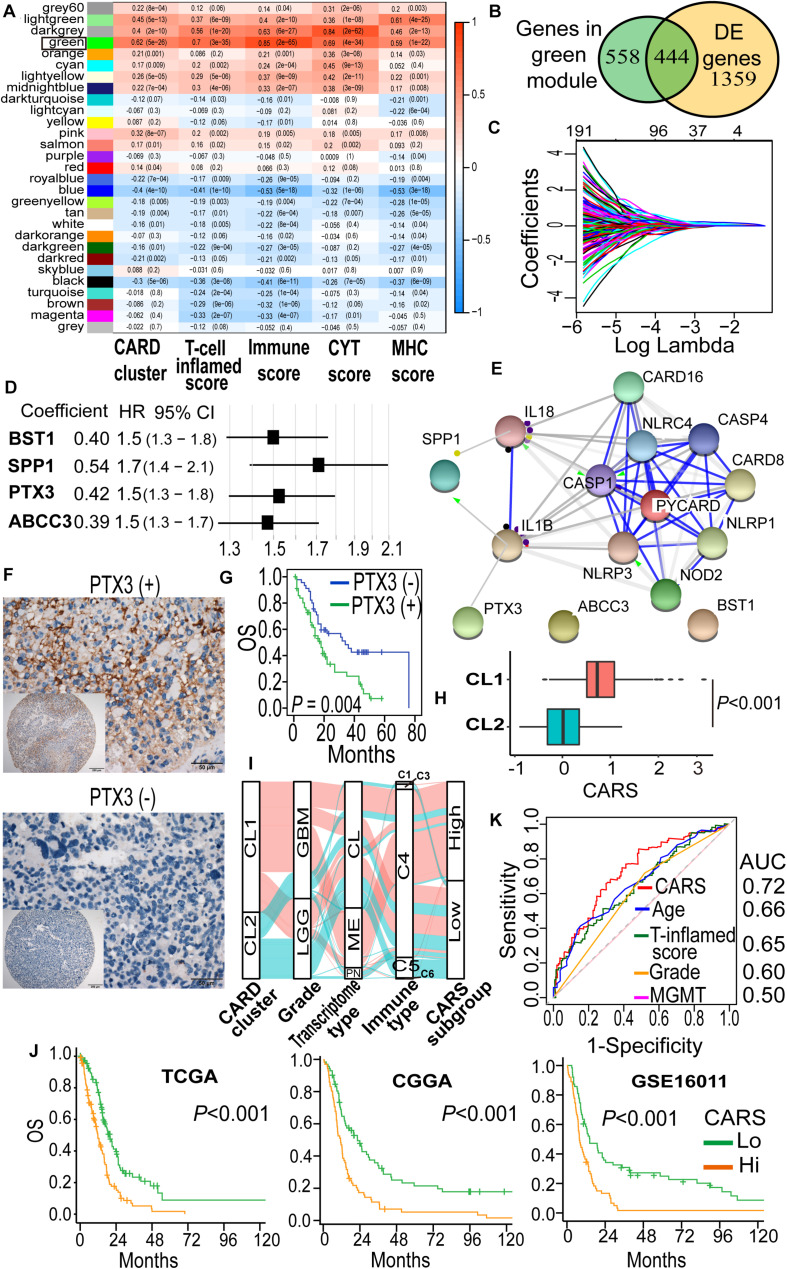
Development of a CARD-associated risk score (CARS) with prognostic significance. **(A)** WGCNA revealed the correlations of gene modules with CARD clusters and immune phenotypes. Coefficient and *P* value of correlations were depicted in heatmap. **(B)** Venn plot shows the overlap of module genes and differently expressed genes. **(C)** LASSO penalized regression identified four genes with optimal prognostic contributions. **(D)** Coefficient and hazard ratio of the four identified genes in the Cox regression model. **(E)** PPI network indicated interactions among the identified genes. **(F)** Representative images of IHC staining for PTX3 expression in IDH-wt glioma samples from SYSUCC hocort. **(G)** Kaplan–Meier analysis of overall survival according to the levels of PTX3 expression. **(H)** Comparison of CARS between CL1 and CL2. **(I)** Alluvial diagram showed the changes of CARD clusters, glioma molecular subtype, and CARS subgroup. **(J)** Kaplan–Meier curves between high- and low-risk groups in TCGA, CGGA, and GSE16011 cohorts. **(K)** ROC curve analyses of CARS, T cell inflamed score and clinical factors.

To identify hub genes that are functionally linked to CARD clusters, WGCNA was performed to determine the co-expression modules. As shown in Figure 3A, transcripts in the green module displayed strong positive correlation with CARD clusters, as well as immune cell infiltration and cytotoxic T cell function (Figure 3A). Approximately half of the transcripts in the green module were differentially expressed between the two CARD clusters (Figure 3B). The 444 overlapped genes were thus used for least absolute shrinkage and selection operator (LASSO) penalized Cox proportional hazards regression to identify genes with best prognostic contribution in IDH-wt glioma (Figure 3C). A CARD-associated gene signature containing four genes (*SPP1, PTX3, ABCC3*, and *BST1*) with prognostic relevance was obtained (Figure 3D). Their expression was highly correlated with CARD-containing genes (Supplementary Figure 1A), and PPI network revealed underlying interactions (Figure 3E). To the survival significance of the CARD-associated genes, we investigated the protein level of one of the four genes, PTX3, in 103 IDH-wt glioma samples from the SYSUCC corhort by using TMA and IHC methods (Figure 3F). We found that the high expression of PTX3 was associated with a decreased OS (Figure 3G), which is consistent with the results of the tranciptional analysis from the TCGA dataset.

Then, a CARS was calculated for each patient using a formula based on the expression levels of these four CARD-associated genes weighted by their regression coefficients in multivariate analysis:

Riskscore=(0.279expression*ofSPP1)+(0.193*expressionofPTX3)+(0.114expression*ofBST1)+(0.118expression*ofABCC3)

The distribution of risk score and the corresponding expression levels of these genes are shown in Supplementary Figures 1B–D. Higher CARS was observed in CL1 as compared with CL2 (Figure 3H). We segregated IDH-wt gliomas into high- and low-risk groups with the median CARS value. We found that GBM and mesenchymal subtypes were enriched in the high-risk group. According to the immune subtype classification proposed by Thorsson et al. (2019), we found that gliomas in high-risk group were dominantly attributed to the C4 subtype whereas the low-risk group had more tumors in the C5 subtype (Supplementary Figure 2). An alluvial diagram was used to visualize the attribute changes of individual samples (Figure 3I). More importantly, patients in the high-risk group exhibited significantly worse outcomes compared with those in the low-risk group (Figure 3J). The prognostic significance of CARS was validated in two additional independent datasets from the CGGA and GSE16011 cohorts (Figure 3H). Multivarite analyses also confirmed the independent prognostic value of the CARS (Table 1). Furthermore, CARS had a higher area under the curve (AUC) in receiver operating characteristic (ROC) analysis as compared with T cell inflamed score and clinical factors such as age, histological grade, and MGMT promoter status, indicating its superiority for prognosis–prediction (Figure 3K).

**TABLE 1:** Univariate and multivariate Cox regression analyses for overall survival of IDH-wt glioma patients in public datasets.

Variables	Univariate analysis			Multivariate analysis		
	HR	95% CI	*P* value	HR	95% CI	*P* value
**TCGA cohort (*n* = 231)**						
Risk score	2.84	2.05–3.93	<0.001	2.98	2.04–4.35	<0.001
Age	1.03	1.02–1.05	<0.001	1.03	1.01–1.04	0.001
Gender (Male/Female)	1.30	0.94–1.80	0.115	–	–	–
Grade (GBM/LGG)	2.34	1.67–3.28	<0.001	1.23	0.80–1.88	0.342
RT (yes/no)	0.57	0.38–0.87	0.009	0.27	0.17–0.42	<0.001
**MGMT**						
Unmethylated	ref			–	–	–
Methylated	1.02	0.71–1.45	0.918	–	–	–
Unknown	1.47	0.96–2.25	0.078	–	–	–
**CGGA cohort (*n* = 147)**						
Risk score	2.17	1.57–3.93	<0.001	1.85	1.32–2.60	<0.001
Age	1.02	1.00–1.03	0.029	1.01	1.00–1.03	0.102
Gender (Male/Female)	1.30	0.89–1.90	0.176	–	–	–
Grade (GBM/LGG)	2.17	1.57–3.00	<0.001	1.77	1.14–2.73	0.011
**RT (yes/no)**						
No	ref			–	–	–
Yes	0.77	0.48–1.23	0.277	–	–	–
Unknown	1.79	0.76–4.21	0.183	–	–	–
**MGMT**						
Unmethylated	ref			–	–	–
Methylated	0.88	0.61–1.30	0.540	–	–	–
Unknown	0.43	0.14–1.38	0.158	–	–	–
**GSE16011 cohort (*n* = 126)**						
Risk score	2.76	1.87–4.07	<0.001	1.58	1.04–2.39	0.033
Age	1.04	1.03–1.06	<0.001	1.03	1.02–1.05	<0.001
Gender (Male/Female)	0.96	0.63–1.45	0.839	–	–	–
Grade (GBM/LGG)	3.91	2.41–6.34	<0.001	2.50	1.46–4.29	0.001
RT (yes/no)	1.10	0.71–1.69	0.672	–	–	–

*Risk score and age were evaluated as continuous variables.HR, hazard ratio; CI, confidence interval.*

### High CARS Indicates the Activation of Adaptive Immunity

**FIGURE 4:**
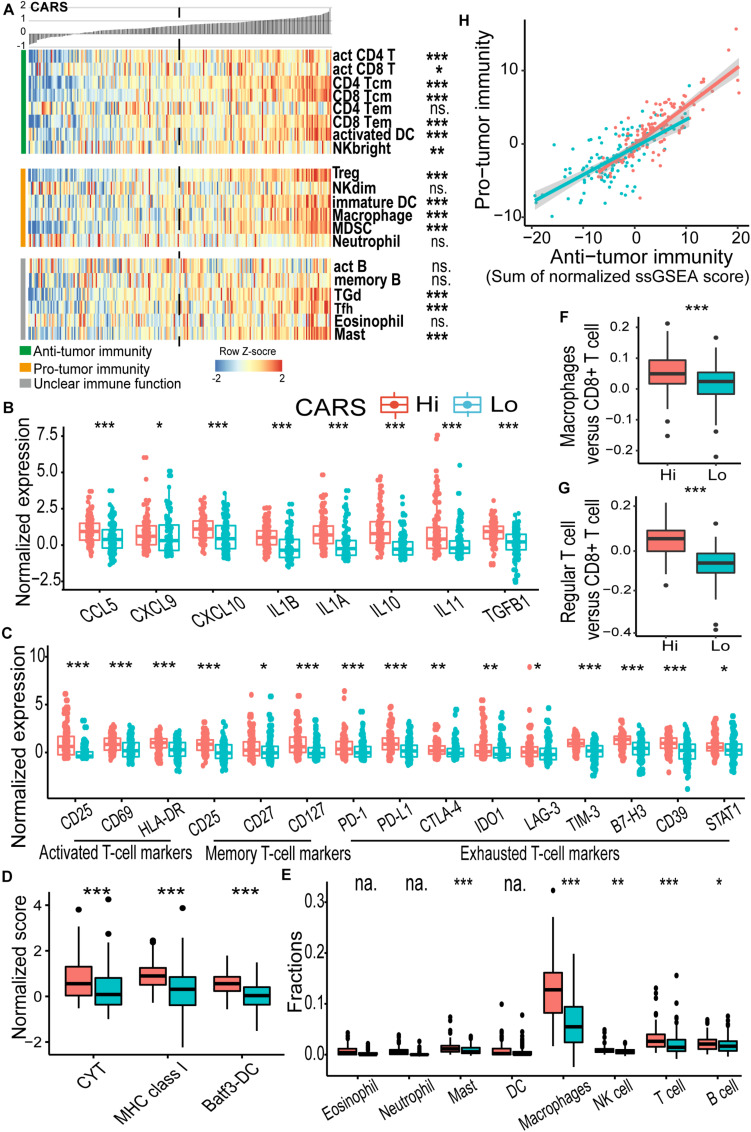
CARS indicates distinct immune landscape in TCGA IDH wild-type gliomas. **(A)** Heatmap showed the ssGSEA score of each immune cell populations between high- and low-risk groups. Immune cells were categorized into anti-tumor immunity, pro-tumor immunity, and unclear immune function. **(B)** Comparisons of expressions of cytokines; **(C)** genes of T cell biomarkers; **(D)** normalized score of CYT, MHC, and Batf3-DC gene sets; **(E)** fractions of infiltrating leukocyte estimated by using CIBERSORT algorithm. **(F,G)** Ratio of macrophages versus CD8+ T cell and regular T cell versus CD8+ T cell between high- and low-risk groups. **(H)** Correlation between infiltrated immune cells for anti- and pro-tumor immunity. Comparison was not implemented when median of cell fraction is less than 0.001. *P* values: *, <0.05; **, <0.01; ***, <0.001; ns., not significant; na., not applicable.

To further unveil the difference of tumor microenvironment (TME) in the high- and low-risk glioma groups, ssGSEA was employed to profile the level of immune cell infiltration and immune cell function in each group. We found a significant difference in major cell components associated with anti-tumor immunity in the two groups (Figure 4A and Supplementary Figure 3). Of note, TME of the high-risk group displayed a prominent T cell infiltration at baseline. We found that biomarkers for activated and memory T cells (Figure 4C, left and middle panels), as well as elevated cytolytic activity (Figure 4D), were dominantly enriched in the high-risk group, indicating the pre-existing adaptive immune response. It has been suggested that the recruitment and priming of effector T cells rely on chemotaxis and the presence of licensed professional antigen-presenting cell populations (Sanchez-Paulete et al., 2017). We showed that significant upregulation of chemokines, including CCL5, CXCL9, and CXCL10, that are pivotal for the intratumoral trafficking of effector T cells was observed in the high-risk group (Figure 4B). In addition, gliomas in the high-risk group had an elevated expression of genes involved in APC activation and function, including activated DC (Figure 4A), MHC-I class, and Batf3-DC signature, which represent cross-presentation of antigen (Figure 4D). Infiltrating cells that enhance innate immune response such as CD56^bright^ natural killer (NK) cells were also enriched (Figure 4A). Collectively, these results indicated that the high-risk glioma group had high CARS and exhibited T cell inflamed features with an increased pre-existing immunological activity.

### High CARS Is Associated With T Cell Exhaustion and a Profound Immunosuppressive Microenvironment

As demonstrated above, patients in the high-risk group had a decreased survival despite the presence of cytotoxic T cells. The impaired function of inflamed T cells might partly account for this paradox. We found that multiple checkpoint receptors and their ligands such as PD-1, PD-L1, CTLA-4, LAG-3, TIM-3, IDO1, and CD39, as well as other immune inhibitory molecules including STAT1, were significantly upregulated in the high-risk group (Figure 4C, right panel) and thus might lead to inhibited function of T cells. The hyporesponsive T cells failed to incite effective anti-tumor immunity to maintain a durable tumor control. Moreover, the immunosuppressive microenvironment has been linked to the oncogenesis and tumor progression. Here, CIBERSORT deconvolution method revealed that macrophages dominantly infiltrated in gliomas of both groups (Supplementary Figure 4A and Figure 4E), whereas the proportion of overall and immunosuppressive M2-type macrophage (Figure 4E and Supplementary Figure 4B) as well as the macrophage/T cell ratio (Figure 4F) was higher in the high-risk group. Similarly, enrichment of regulatory T cells (Treg; Figure 4A) and an elevated Treg/T cell ratio (Figure 4G) were also observed in the high-risk group. In addition, we found an accumulation of other immune inhibitory cellular components (e.g., immature DC, CD56^dim^ NK cell, MDSC; Figure 4A) and cytokines (e.g., IL10, IL11, and TGFB1; Figure 4B) in the high-risk group. Consistent findings were also observed in the CGGA cohort (Supplementary Figure 3). In addition, we evaluated the immune homeostasis in these two groups by looking at the interaction between cells with anti-tumor or pro-tumor activities. Pearson’s correlation analysis revealed that the abundance of immune cells of these two populations was positively associated with each group (Figure 4H), indicating the activation of immunosuppressive components in the TME.

### CARS Is Associated With the Disturbance of Oncogenic Pathways in Gliomas

**FIGURE 5:**
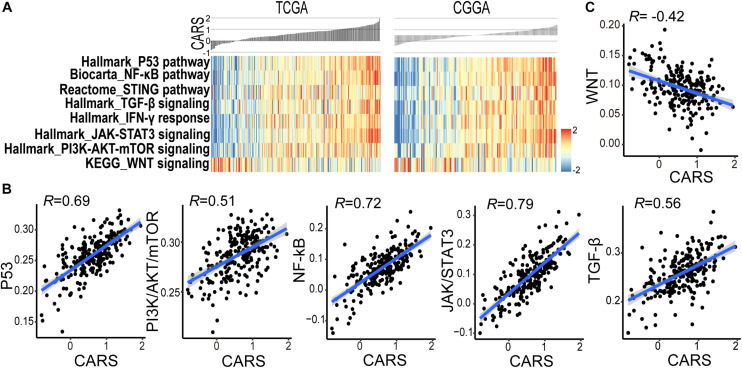
CARS associates with disturbed oncogenic pathways. **(A)** Heatmap showed the ssGSEA score of the signaling pathway from the MSigDB database between high- and low-risk groups in TCGA (left) and CGGA (right) cohorts. **(B,C)** Spearman correlations between CARS and ssGSEA score of oncogenic pathways.

**FIGURE 6:**
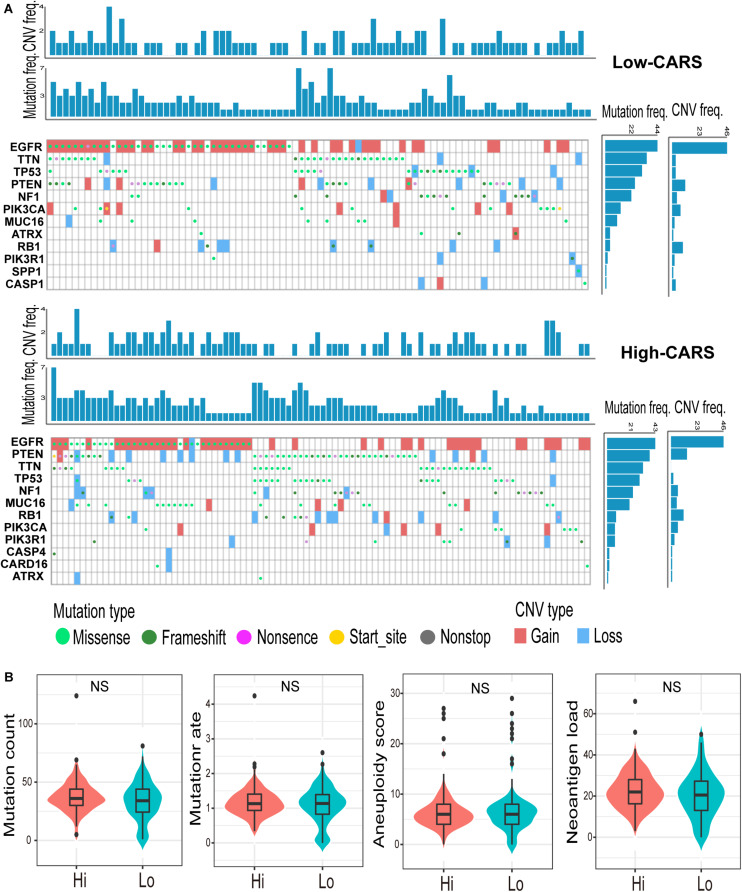
Genomic alterations in the high- and low-risk group. **(A)** Waterfall plot of glioma somatic mutation and copy-number variation (CNV) in the high- (up) and low-risk group (down). Each column represented individual samples. The upper barplot showed mutation and CNV loads in each sample, and the right barplot indicated mutation and CNV frequencies in each gene. **(B)** Comparisons of non-silence mutation count, mutation rate, aneuploidy score, and neoantigen load between high- and low-risk groups. ns., not significant.

Accumulating evidence suggests that cancer-cell-intrinsic properties play a critical role in shaping the tumor immune microenvironment (Wellenstein and de Visser, 2018). In our study, we found that the major oncogenic pathways, such as PI3K/Akt/mTOR, JAK/STAT3, NF-κB, and TGF-β, were upregulated in the high-risk group (Figures 5A,B and Supplementary Figure 5), and activation of the WNT/β-catenin pathway was evident in the low-risk group (Figure 5C and Supplementary Figure 5). We also analyzed the genomic alterations in both groups. Significant enrichments of mutations of PTEN, PIK3R1, RB1, and MUC16 were observed in the high-risk group (Figure 6A and Supplementary Figure 6). There was no statistical difference in overall tumor mutation, aneuploidy, and neoantigen loads between the two groups (Figure 6B).

### A High CARS Implicates an Improved Outcome of Anti-PD-1 Immunotherapy for IDH-wt Gliomas

**FIGURE 7:**
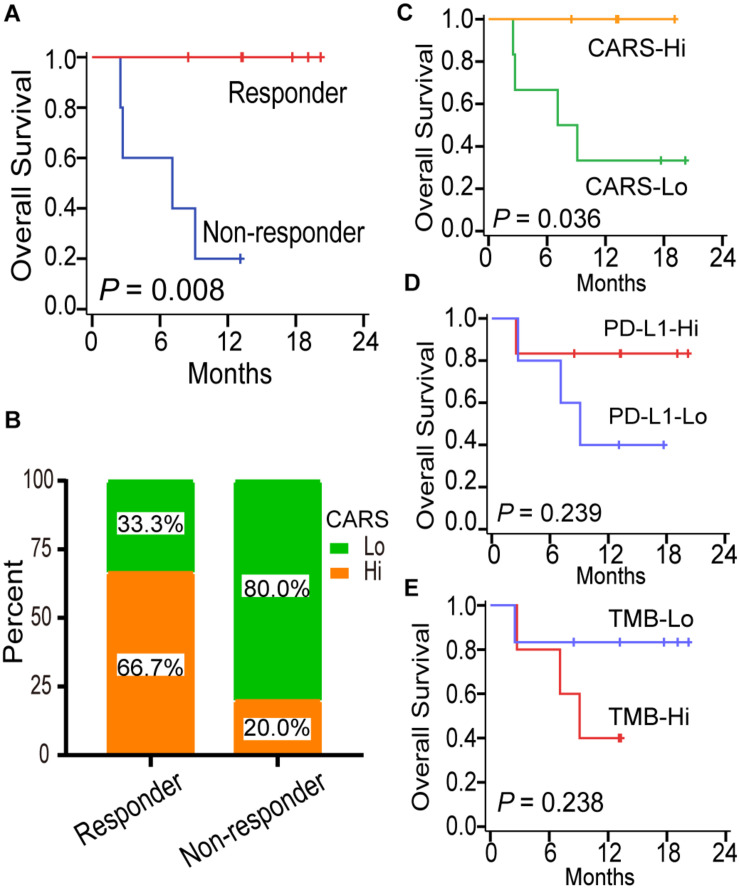
CARS predicts survival benefit for glioma patients treated with anti-PD-1 therapy. **(A)** Kaplan–Meier curves between immunotherapy-responder and non-responder. **(B)** Distribution of high- and low-risk group in immunotherapy-responder and non-responder. **(C–E)** Kaplan–Meier curves between distinct subgroups of CARS, PD-L1, and TMB based on their median levels.

Although several multi-gene expression signatures were developed to delineate tumor immune microenvironment and implied to be candidate biomarkers for predicting ICI treatment response in gliomas, none of them have been validated (Zhu et al., 2019; Wu et al., 2020). Here, we investigated the predictive performance of CARS by using recently published data on transcriptome as well as clinical data of patients with recurrent IDH-wt gliomas under anti-PD-1 therapy (Zhao et al., 2019). In that study, responders, who were defined as radiologically stable for at least 6 months and/or having very few to no tumor cells in surgical tissues after anti-PD-1 therapy, had a favorable prognosis compared to non-responders (Figure 7A). We showed that recurrent gliomas with a high CARS were enriched in responders of anti-PD-1 therapy (Figure 7B). Moreover, we also found that an increased CARS was associated with a prolonged OS after anti-PD-1 therapy (Figure 7C). The median OS was not reached in patients with a high CARS and 8.1 months for those with a low CARS (HR: 0.12; 95% CI: 0.02–0.88; *P* = 0.036). In contrast, PD-L1 expression (Figure 7D) and tumor mutation burden (Figure 7E) failed to predict the prognosis of ICI in IDH-wt gliomas. Our data suggested that CARS had the capability to identify glioma patients who are more likely to benefit from anti-PD-1 therapy.

## Discussion

Large randomized trials have failed to recapitulate the efficacy of immune checkpoint blockade in unselected patients with gliomas. The heterogeneity of glioma immune microenvironment and the lack of predictive biomarkers for responsiveness to ICI may account for these clinical outcomes. Although several signaling pathways and cellular mechanisms have been explored in immune modulations in gliomas (Quail and Joyce, 2017; Cai et al., 2018; Zha et al., 2020), none of them have been successfully translated to the clinic to identify patients who will benefit from immunotherapy. Here, by integrating the TCGA dataset, publicly available transcriptional profiles and clinical outcome data, we developed a CARD-associated risk scoring system, which integrated with the heterogeneity of immune microenvironment and molecular landscape, as well as the difference in prognosis and responsiveness to ICI in IDH-wt gliomas.

Glioma is generally considered as a “cold” tumor with less lymphocyte infiltration. A pan-cancer immunogenomic classifier attributed gliomas to C4 (lymphocyte depleted) or C5 (immunologically quiet) subtypes, and both are characterized by the paucity of tumor-infiltrating lymphocytes (TILs) (Thorsson et al., 2019). In fact, TILs can be frequently identified in gliomas, but the extent varies greatly among tumors. We demonstrated that TILs were more evident in high-risk gliomas. The mechanisms that underpin the accumulation or exclusion of lymphocytes within TME have not been fully understood. Results from previous studies suggested that tumor neoantigens are crucial to the recruitment of lymphocytes (Becht et al., 2014). However, no difference was found in neoantigen load between the high- and low-risk group in the present study. Spranger et al. revealed that upregulation of the oncogenic β-catenin pathway suppressed the production of crucial chemokine CCL4 and subsequently prevented recruitment of intratumoral CD103^+^ DCs, followed by diminishing T lymphocytes in melanomas (Spranger et al., 2015). In our study, activation of β-catenin signaling pathway as well as reduction in DCs and cross-presentation process was observed in low-risk gliomas, which may contribute to the exclusion of T cells.

The infiltration of lymphocytes has been reported to imply an adaptive immunity against tumor progression and positively impact clinical survival in a variety of tumor types including gliomas (Lohr et al., 2011; Sanchez-Canteli et al., 2020). However, conflicting evidences exist. Rutledge et al. (2013) quantified TILs in 171 GBMs from the TCGA database and found that the number of TILs is correlated with specific histopathological features but not with survival. Zhai and colleagues (Zhai et al., 2017) showed that infiltrating T lymphocytes in GBMs contributed to the worse prognosis by directly inducing IDO1 expression. In this study, we found that high-risk IDH-wt glioma was enriched with TILs but had a decreased overall survival. There are several reasons that may account for the inconsistency. Firstly, various approaches were employed to characterize lymphocyte infiltration. Here, we inferred TILs by using the transcription-based algorithms. In some other studies, TILs were determined through visual evaluation of stained slides. The accuracy of histopathological estimation was affected by factors such as the inter-observer bias and heterogeneous distribution of T cells in tissue sections and slides (Khoury et al., 2018). Secondly, the difference in the sample size among studies may also result in the discrepancy. Moreover, we found that the prominent lymphocytes infiltration was accompanied by the dominance of M2-type macrophage as well as abundant negative regulators (e.g., CTLA-4 and IDO1), which constituted a microenvironment preferentially toward pro-tumor activities and fostered tumor progression.

T cell exhaustion was initially described in a mouse model of chronic viral infection, where the presence of persistent antigen diminished the pathogen-specific T cell response. Cancer also exploits this mechanism to induce immune escape. Our findings demonstrated that T cells in high-risk glioma shared key features of T cell exhaustion such as the expression of co-inhibitory receptors including PD-1, LAG-3, and TIM-3, as observed in chronic infections. Traditionally, T cell exhaustion was defined as a terminal differentiation state with the characteristics of anergy, which suppresses the immunosurveillance against tumorigenesis. However, accumulating evidence suggested that exhausted T cells are not fully inert but retain the expansion capacity and the capability to produce chemokines (Li et al., 2019). Furthermore, intratumoral exhausted T cells exhibit cytotoxic and effector potential. Davidson et al. (2019) demonstrated that PD1^+^CD8^+^ T cells in gliomas had elevated TCR clonality and decreased diversity, reflecting the activation of specific anti-tumor machinery within this cell population. In line with those findings, we revealed that T cells in high-risk glioma also displayed activated features by increased expression of effector T cell genes (e.g., CD25, CD69, and HLA-DR), as well as cytolytic biomarkers. The pre-existing exhausted T cells in high-risk glioma partially sustain immunocompetence and are the targets of checkpoint-based immunotherapy.

Multi-gene signatures have been developed to predict treatment benefits of ICI in a variety of cancers. An 11-immune-related gene signature was found to be prognostic for patients with cervical cancer when treated with ICI (Yang et al., 2019). Ayers et al. (2017) confirmed the predictive usefulness of an IFN-γ-related mRNA profile for the response to pembrolizumab in melanomas. However, no predictive transcriptional biomarker has been established with validation in gliomas due to the scarcity of both pre-treatment tumor samples with sequencing data and associated clinical outcomes. In our study, we evaluated the predictive value of CARS in a recently published cohort of glioma patients treated with PD-1 inhibitors (Zhao et al., 2019). We showed that an increased CARS was dominantly identified in ICI responders and predicted a prolonged survival, whereas PD-L1 transcription and TMB were not associated with clinical outcomes. Our data suggested that CARS can be employed to identify glioma patients who will obtain the most benefit from PD-1 checkpoint blockade, but validation in clinical trials with large sample size is warranted.

Our study also suggests different mechanisms underlying the primary resistance to ICI among IDH-wt gliomas. High-risk glioma showed evidence of pre-existing T cells that can be potentially reinvigorated by the blockade of PD-1. However, a broad set of immunosuppressive mechanisms are active in the TME. Overexpression of immune inhibitory molecules such as IDO1 and TIM-3, together with higher proportions of M2-type macrophage, Treg, and MDSCs, impeded the antitumor immune response triggered by ICI. Accordingly, antagonism of immunosuppressive signaling other than the PD-1/PD-L1 axis is feasible to increase the activity of checkpoint-based therapies in the high-risk group. On the other hand, the lack of priming signals and T cell infiltration at baseline in low-risk glioma appears to correlate with ICI insensitivity. Detonating the immunogenicity with tumor- and dendritic cell-based vaccination or oncolytic viruses will enhance the anti-PD-1 efficacy in this subset of tumors.

## Conclusion

Our study developed a CARS, which categorized IDH-wt gliomas into two groups with distinct immune and molecular characteristics. An increased CARS is associated with a profound immunosuppressive microenvironment with pre-existing adaptive immunity. Patients in the high-risk group had a reduced OS under standard care but tended to benefit from checkpoint immunotherapy. We believe that our findings will enhance the understanding of heterogeneity in the tumor immune microenvironment and contribute to tailoring immunotherapy for IDH-wt gliomas.

## Data Availability Statement

The datasets presented in this study can be found in online repositories. The names of the repository is the Research Data Deposit public platform (www.researchdata.org.cn), with the approval number as RDDB2021001075.

## Ethics Statement

The studies involving human participants were reviewed and approved by the Ethics Committee of Sun Yat-sen University Cancer Center. Written informed consent to participate in this study was provided by the participants’ legal guardian/next of kin.

## Author Contributions

KS, YM, and ZC designed this study. WH, ZH, and HD performed the experiments. DL, PZ, XW, SZ, XJ, and JZh analyzed the data. DL, XL, and CG drafted this manuscript. KS, JW, and JZe revised the manuscript. All authors approved the publication of the manuscript.

## Conflict of Interest

The authors declare that the research was conducted in the absence of any commercial or financial relationships that could be construed as a potential conflict of interest.

## Footnotes

1 https://xenabrowser.net

2 http://www.cgga.org.cn

3 https://www.ncbi.nlm.nih.gov/geo

4 https://string-preview.org

5 https://www.immport.org
