# Supplementary material for: CARD-Associated Risk Score Features the Immune Landscape and Predicts the Responsiveness to Anti-PD-1 Therapy in IDH Wild-Type Gliomas
Source: Front Cell Dev Biol. 2021 Mar 19;9:653240. doi: 10.3389/fcell.2021.653240 (PMC8009185; doi:10.3389/fcell.2021.653240)
Supplement: Supplementary file 1 [file Image_1.PDF]

Figure S1. Development of a risk scoring system with four CARD-associated genes. (A) Spearman correlation among the expressions of CARD-associated genes. (B) Distribution of groups based on the risk score. (C) Survival status of patients in different groups. (D) Heatmap of expression profiles of the four identified genes.

Figure S2. Proportion of tumors stratified by pathology or molecular subtypes between different risk groups. GBM, glioblastoma; LGG, lower-grade gliomas; CL, classical; ME, mesenchymal; NE, neural; PA, pilocytic astrocytoma. C1: wound healing; C2: IFN-γ dominant; C3: inflammatory; C4: lymphocyte depleted; C5: immunologically quiet; C6: TGF-β dominant.

Figure S3. CARD-associated risk score indicates distinct immune landscape in gliomas from CGGA cohort. (A) Heatmap showed ssGSEA score of each immune cell populations between high- and low-risk groups. (B) Comparisons of normalized score of cytolysis activity (CYT), MHC and Batf3-DC gene sets, (C-D) expressions of cytokines and T-cell markers between high- and low-risk groups.

Figure S4. Fractions of infiltrating leukocyte in TCGA IDHwt gliomas estimated by using CIBERSORT algorithm.
(A) Overview of leukocyte infiltration. Samples were ranked by their CARS value. Immune cells were categorized into B cells, dendritic cells (DC), eosinophils, macrophages, mast cells, neutrophils, natural killer cells and T cells.
B cells include B.cells.naive, B.cells.memory and Plasma.cells.
DC include DC. resting and DC. activated.
Macrophages include Monocytes, Macrophages.M0, Macrophages.M1 and Macrophages.M2.
Mast cells include Mast.cells.resting and Mast.cells.activated.

NK include NK.cells.resting and NK.cells.activated.

T cells include T.cells.CD4.naive, T.cells.CD4.memory.resting, T.cells.CD4.memory.activated, T.cells.follicular.helper,, Tregs, T.cells.gamma.delta and T.cells.CD8.

(B) Macrophage M2 is the major type of macrophages infiltrating in gliomas and is significantly increased in high-risk group as compared with low-risk group.

Figure S5. Spearman correlations between CARS and ssGSEA score of oncogenic pathways in CGGA cohort.

Figure S6. Comparison of the mutation frequency of common mutated genes between high- and low-risk groups. Chi-squared test were used for comparison.

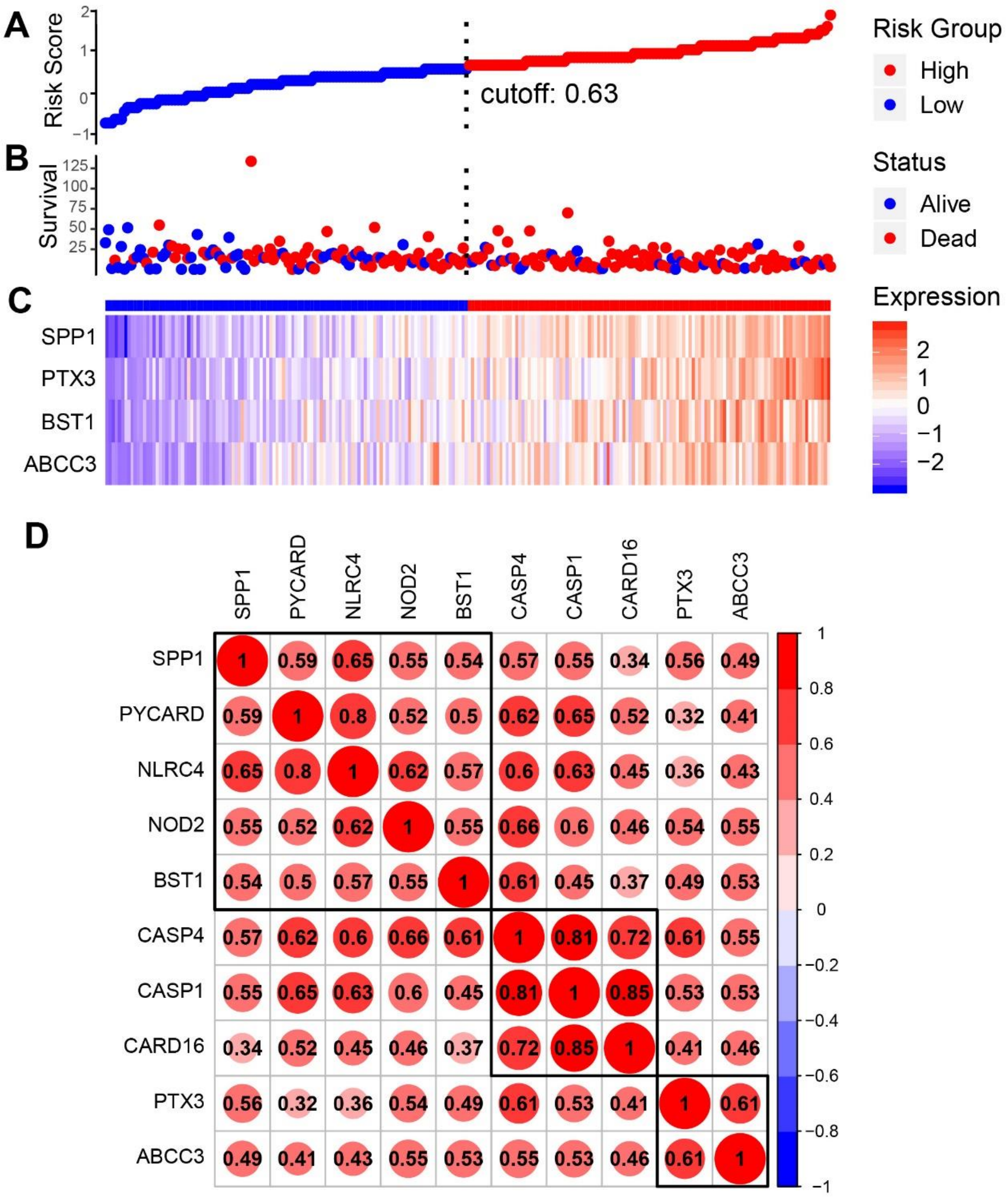
A
Risk Score
cutoff: 0.63
Risk Group
High
Low
B
Survival
Status
Alive
Dead
C
SPP1
PTX3
BST1
ABCC3
Expression
D
SPP1
PYCARD
NLRC4
NOD2
BST1
CASP4
CASP1
CARD16
PTX3
ABCC3

Figure S1

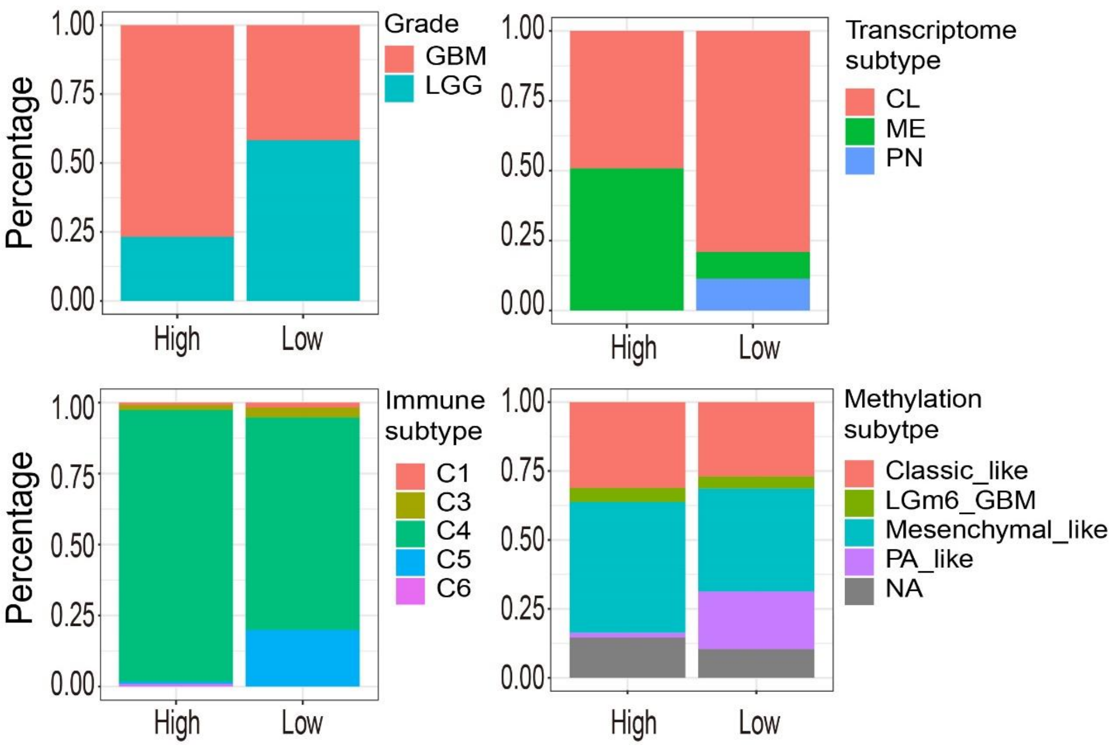

Percentage
1.00
0.75
0.50
0.25
0.00
High
Low
Grade
GBM
LGG
Transcriptome subtype
CL
ME
PN
Immune subtype
C1
C3
C4
C5
C6
Methylation subytpe
Classic_like
LGm6_GBM
Mesenchymal_like
PA_like
NA

Figure S2

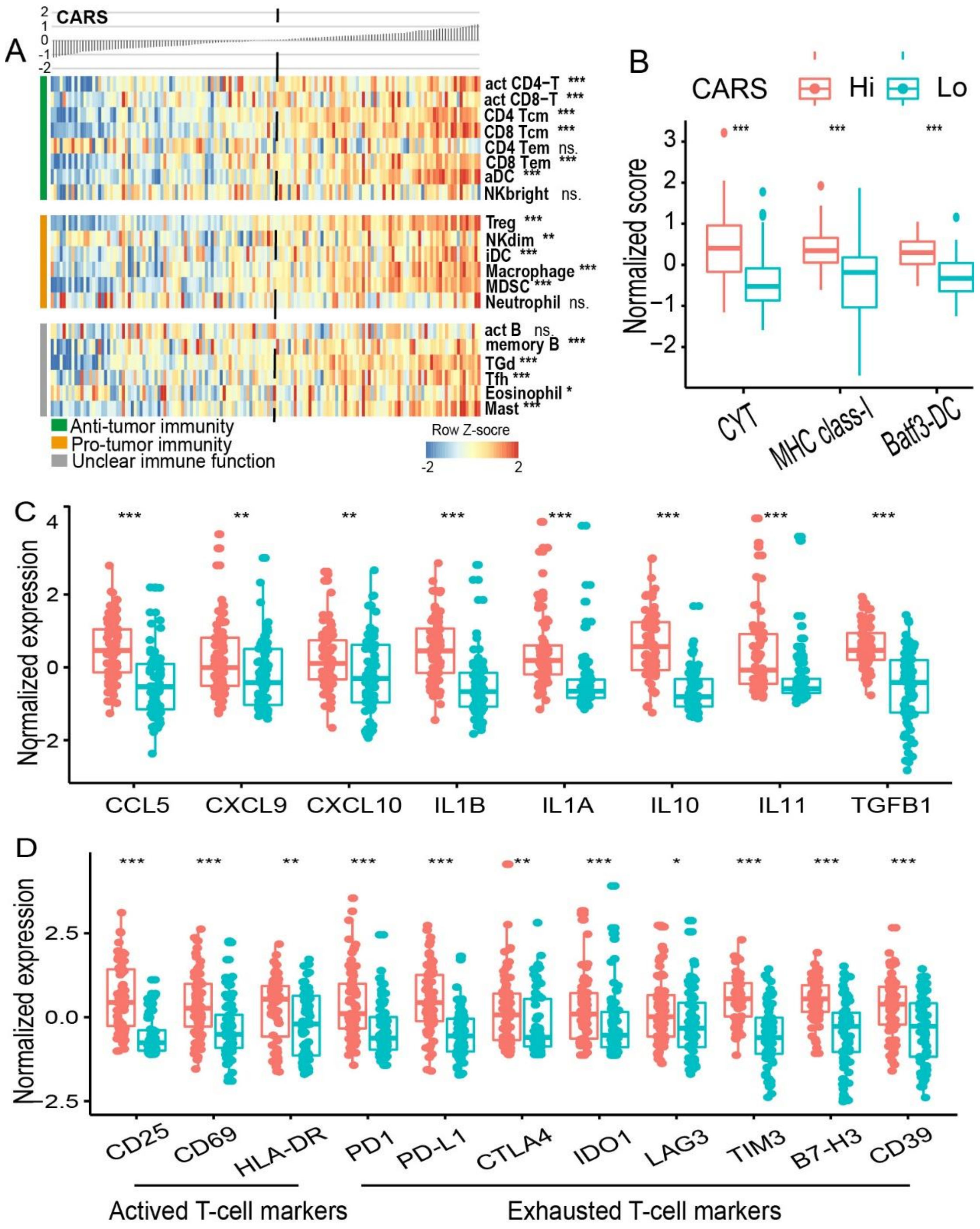
A
CARS
act CD4−T ***
act CD8−T ***
CD4 Tcm ***
CD8 Tcm ***
CD4 Tem ns.
CD8 Tem ***
aDC ***
NKbright ns.
Treg ***
NKdim **
iDC ***
Macrophage ***
MDSC ***
Neutrophil ns.
act B ns.
memory B ***
TGd ***
Tfh ***
Eosinophil *
Mast ***
Anti-tumor immunity
Pro-tumor immunity
Unclear immune function
Row Z-socre
B
CARS Hi Lo
Normalized score
CYT
MHC class-I
Batf3-DC
C
Normalized expression
CCL5
CXCL9
CXCL10
IL1B
IL1A
IL10
IL11
TGFB1
D
Normalized expression
CD25
CD69
HLA-DR
PD1
PD-L1
CTLA4
IDO1
LAG3
TIM3
B7-H3
CD39
Actived T-cell markers
Exhausted T-cell markers

Figure S3

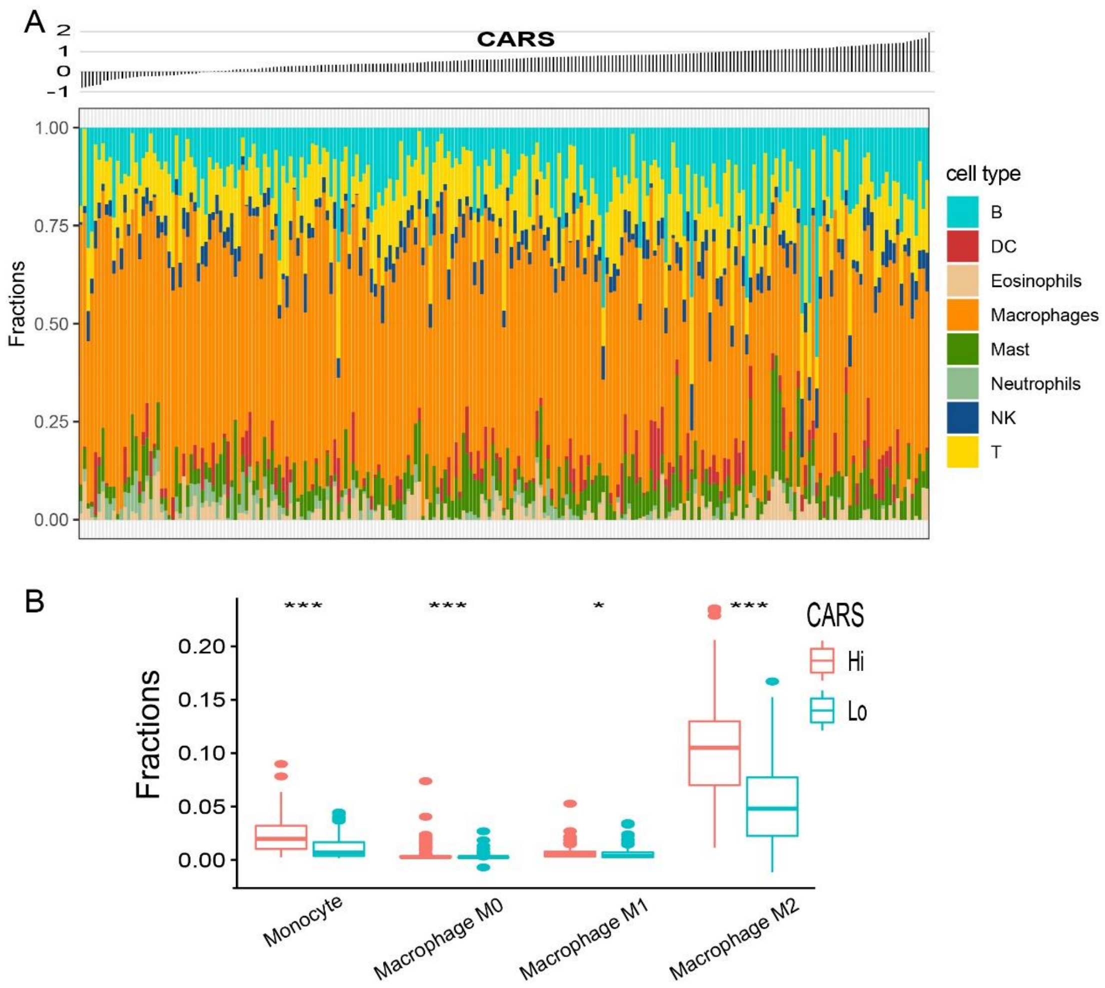
A
CARS
2
1
0
-1
1.00
0.75
0.50
0.25
0.00
Fractions
cell type
B
DC
Eosinophils
Macrophages
Mast
Neutrophils
NK
T
B
***
***
*
***
0.20
0.15
0.10
0.05
0.00
Fractions
CARS
Hi
Lo
Monocyte
Macrophage M0
Macrophage M1
Macrophage M2

Figure S4

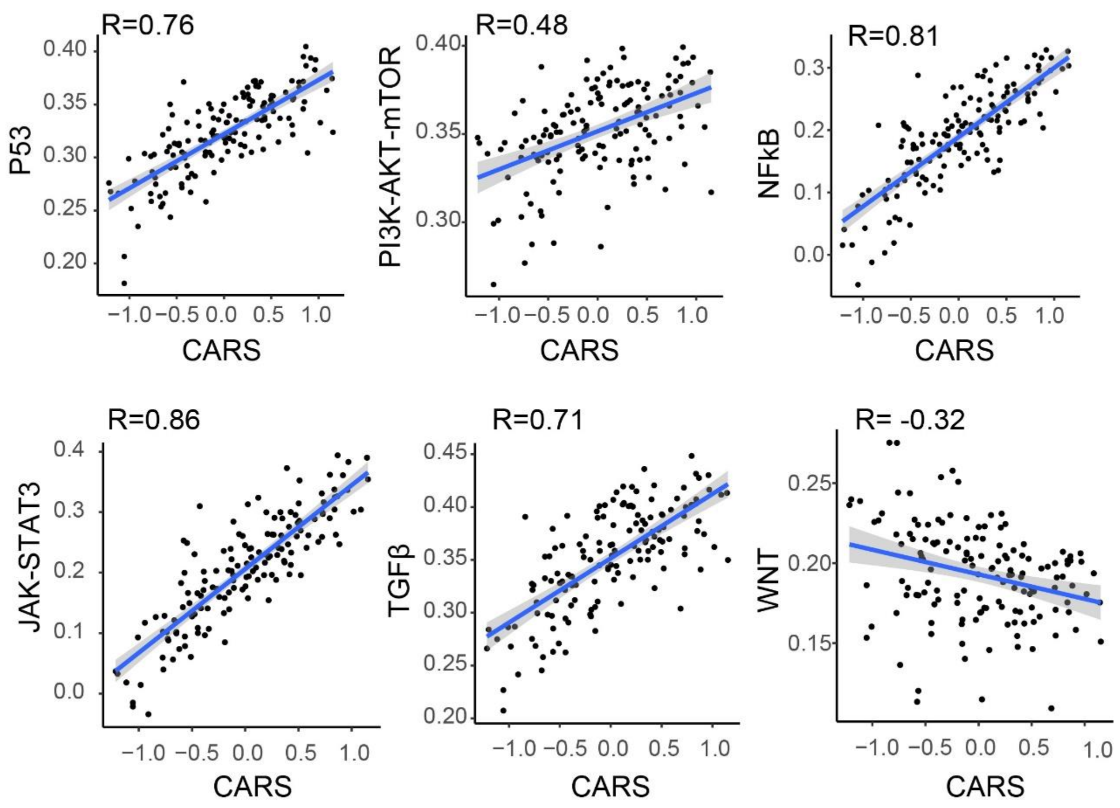
R=0.76
P53
0.40
0.35
0.30
0.25
0.20
−1.0 −0.5 0.0 0.5 1.0
CARS
R=0.48
PI3K-AKT-mTOR
0.40
0.35
0.30
−1.0 −0.5 0.0 0.5 1.0
CARS
R=0.81
NFkB
0.3
0.2
0.1
0.0
−1.0 −0.5 0.0 0.5 1.0
CARS
R=0.86
JAK-STAT3
0.4
0.3
0.2
0.1
0.0
−1.0 −0.5 0.0 0.5 1.0
CARS
R=0.71
TGFβ
0.45
0.40
0.35
0.30
0.25
0.20
−1.0 −0.5 0.0 0.5 1.0
CARS
R= -0.32
WNT
0.25
0.20
0.15
−1.0 −0.5 0.0 0.5 1.0
CARS

Figure S5

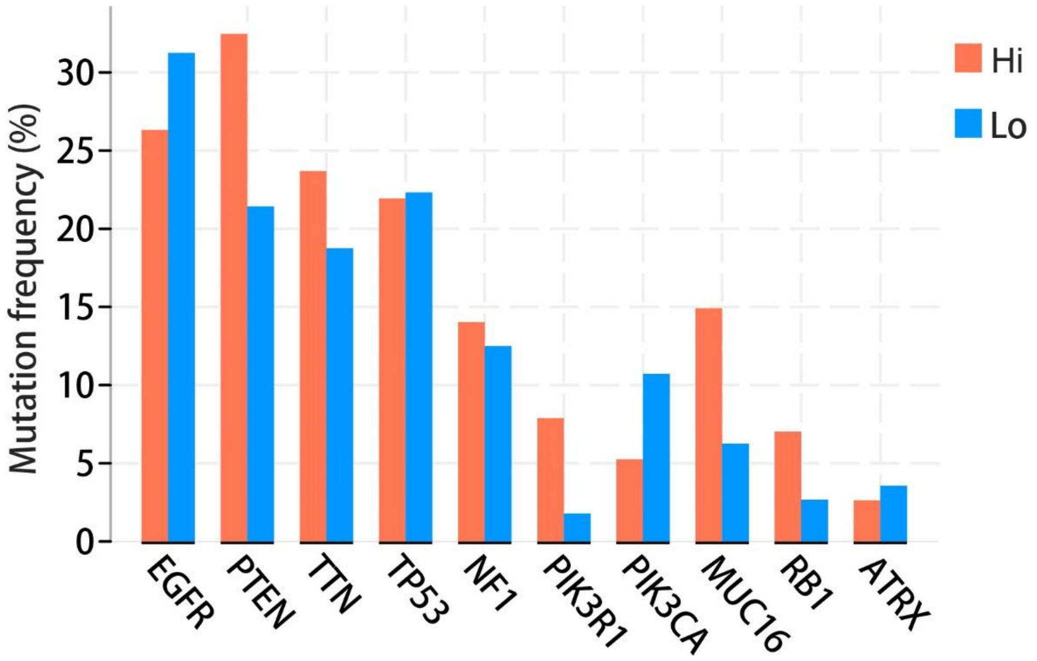
Mutation frequency (%)
30
25
20
15
10
5
0
Hi
Lo
EGFR
PTEN
TTN
TP53
NF1
PIK3R1
PIK3CA
MUC16
RB1
ATRX

Figure S6
