# Supplementary material for: CARD-Associated Risk Score Features the Immune Landscape and Predicts the Responsiveness to Anti-PD-1 Therapy in IDH Wild-Type Gliomas
Source: Front Cell Dev Biol. 2021 Mar 19;9:653240. doi: 10.3389/fcell.2021.653240 (PMC8009185; doi:10.3389/fcell.2021.653240)
Supplement: Supplementary file 2 [file Table_1.DOCX]

**Table S1.** Summary of IDHwt glioma datasets used in the current study

|  | TCGA (n=231) | CGGA (n=145) | GSE16011 (n=126) |
| --- | --- | --- | --- |
| Age (x±S) | 57.9±13.7 | 48.8±12.9 | 52.0±14.5 |
| Gender |  |  |  |
| Male | 139/60.2% | 94/64.8% | 91/72.2% |
| Female | 92/39.8% | 51/35.2% | 35/27.8% |
| Grade |  |  |  |
| GBM | 137/59.3% | 101/69.0% | 90/71.4% |
| LGG | 94/40.7% | 44/31.0% | 36/28.6% |
| MGMT promoter |  |  |  |
| Methylated | 75/32.5% | 47/32.4% | - |
| Unmethylated | 127/55.0% | 93/64.1% | - |
| NA | 29/12.5 | 5/3.4% | - |
| Radiotherapy |  |  |  |
| Yes | 169/73.2% | 11176.6% | 93/73.8% |
| No | 40/17.3% | 27/18.6% | 33/26.2% |
| NA | 224.8% | 7/4.8% | 0/0% |
| Follow-up duration, mo, median (IQR) | 12.6  (5.6~18.7) | 13.5  (8.3~26.6) | 10.8  (6.5~22.2) |
| Overall deaths | 161/69.7% | 122/84.1% | 116/92.1% |
